# Assessment of the needs and demand for psychiatric care among medical students: cross-sectional study

**DOI:** 10.1192/j.eurpsy.2025.1997

**Published:** 2025-08-26

**Authors:** A. Ben Haouala, N. Faouel, E. Abouda, L. Gassab, B. Amamou, A. Mhalla

**Affiliations:** 1 Research Laboratory “Vulnerability to psychotic disorders”, Faculty of Medicine; 2 Psychiatry, University Of Monastir, Monastir, Tunisia

## Abstract

**Introduction:**

Studying medicine is a demanding and time-consuming course of study, which can be a major stress factor and affect quality of life (QOL). Medical students are at high risk of developing mood disorders. This could lead to a need for psychiatric care (PC) that is not necessarily equivalent to the demand.

**Objectives:**

Determine the prevalence of depression, anxiety and stress in medical students, assess their QOL and subjective need for PC.

**Methods:**

This was a descriptive cross-sectional study conducted over a 5-month period in a Tunisian medical school. Sampling was stratified random. Sociodemographic and clinical data were collected using a questionnaire. Depression, anxiety and stress were assessed using the Depression Anxiety and Stress Scale: DASS-21. QOL was assessed using the generic 36-item Short-Form Health Survey (SF-36). Subjective assessment of difficulties and need for help was carried out using the “KIT ELADEB: Lausanne Self-Assessment Scales for Difficulties and Needs”.

**Results:**

The study included 308 students with a median age of 21. Some (18.8%) were smokers, 15.9% drank alcohol, and 5.2% used psychoactive substances (PAS). A Family psychiatric history (PH) was present in 15.4% of cases, and 14.3% of students had personal PH. Median DASS scores were: depression 18 (interquartile range (IQR) = 16-27), anxiety 20 (IQR = 18), and stress 16 (IQR = 42). The rates of extremely severe and severe depression were 27.6% and 12.3% respectively, while 50.6% had extremely severe and 12% severe anxiety. Some 29.2% of students had severe to extremely severe stress. Mean SF-36 scores were 61.19 ± 16.83, referring to the Léan cut-off value (66.7), 58.1% had impaired QOL, 15.9% of participants had consulted a psychiatrist, 78.9% had felt the need to do so, and 59% had foregone such help. Factors associated with severe depression included advanced age (p=0.007), graduate education (p<10-3), PAS use (p<10-3), and PH (p<10-3). Anxiety was predicted by female gender (FG) (p=0.04), SPA use (p=0.03), and PH (p<10-3). Stress was associated with age (p=0.039), course of study (p=0.008), lack of leisure activities (p=0.003), smoking (p=0.004), alcoholism (p=0.009), use of PAS (p=0.003), and PH (p=0.000). Students who consulted a psychiatrist were more likely to have depression (p<10-3), stress (p<10-3), and impaired QOL (p<10-3). Participants who expressed a subjective need to consult a psychiatrist were significantly associated with FG (p<10-3), depression (p<10-3), anxiety (p<10-3), stress (p<10-3), and alterations in the mental health (p=0.008), vitality (p=0.006), social functioning (p=0), and general QOL (p=0.002) dimensions of the SF-36.

**Conclusions:**

The obvious deterioration in the mental health and QOL of medical students, and the mismatch between the need for and demand for PC, should initiate a debate on recommendations to improve the well-being of our future doctors.

**Disclosure of Interest:**

None Declared

